# Biocompatibility of three different root canal sealers, experimental study

**DOI:** 10.1186/s12903-023-03473-2

**Published:** 2023-10-04

**Authors:** Ahmad Alfahlawy, Manar A. A. Selim, Hayam Y. Hassan

**Affiliations:** 1 Endodontic Department, Faculty of Dentistry, Suez Canal University, Ismailia, Egypt; 2 Oral Biology Department, Faculty of Dentistry, Suez Canal University, Ismailia, Egypt; 3 Professor & Chairman of Endodontic Department, Faculty of Dentistry, Suez Canal University, Ismailia, Egypt

**Keywords:** Biocompitability, GuttaFlow Bioseal, Gutta-Percha, Root Canal Filling Materials, Well-Root St, AH plus

## Abstract

**Objectives:**

This study was assessed the biocompatibility of three different root canal sealers (Well-Root St, GuttaFlow Bioseal, and AH-Plus) following implantation in rat subcutaneous tissues, using histopathological immunohistochemical analysis.

**Methods:**

Four groups of eighty-four male rats each underwent subcutaneous dorsal implantation of a polyethylene tube, either empty or filled. Tissues were collected, fixed, and processed for histological analysis after 7, 15, and 30 d. Slides were photographed and digitally processed to identify lymphocytes and macrophages using Cluster of differentiation 3 (CD3) and cluster of differentiation 68 (CD68) markers, respectively. P was set at 0.05, when lymphocyte and macrophage infiltration was compared between groups and observation times using one-way analysis of variance (ANOVA).

**Results:**

Histopathological analysis of all groups revealed an inflammatory reaction followed by the emergence of a fibrous capsule after 7 days. After 30 days, the thickness of the fibrous capsule and the inflammatory response subsided. CD3 staining for immunohistochemical analysis revealed that the AH-Plus group had the highest mean percentage of lymphocyte infiltration at 7 and 15 days, followed by the Well-Root St, GuttaFlow Bioseal, and Control groups. After 30 days, no discernible difference was observed between the groups in terms of the mean percentage of lymphocyte infiltration. After 7, 15, and 30 days, there was a significant difference in the mean percentage of macrophage infiltration across the groups, as demonstrated by CD68 staining. After 7, 15, and 30 days, the AH-Plus group had the highest mean percentage of macrophage infiltration, followed by the Well-Root St. and GuttaFlow Bioseal groups, while the control group had the lowest mean percentage.

**Conclusion:**

All observational periods showed minimal inflammatory reactions to GuttaFlow Bioseal. After subcutaneous tissue implantation in a rat model, the initial inflammatory reactions to Well-Root St and AH-Plus had abated by day 30, and all tested sealers had outstanding biocompatibility.

## Introduction

Correct three-dimensional (3D) cleaning of the root canal system during root canal shaping is crucial for the short- and long-term success of endodontic therapy. Complete 3D obturation of the intricate root canal system will occur next [1].

To prevent germs, pathogens, and fluids from traveling from the coronal to the apical or vice versa, and to relieve pain and infection, the primary objectives of the root canal filling procedure are to restore function and appearance [2].

Therefore, materials used in root canal treatment should be less insoluble to prevent degradation by body fluids [3]. It is well recognized that the extraction of materials used in root canal treatment may have a deleterious impact on the periradicular cell populations.

The entire root canal and surrounding tissues can communicate with each other, in addition to the apical foramen. The root canal and its surrounding tissues (the periodontal ligament and alveolar bone), namely the dentinal tubules, lateral canals, and accessory foramina, have numerous microscopic and macroscopic connections [4].

As a result, tissue fluid can easily enter the root canal system and cause the sealer material to break down, releasing numerous components. When these components are transmitted to nearby tissues, they may cause local periapical inflammatory responses and have negative effects [5, 6].

The requirement that the material should display the correct biological response to the host tissue through a particular application is one of the most crucial aspects of biocompatibility. This idea considers how host, substance, and desired functions are related. A substance is biocompatible if these three elements function together [7].

However, the active components in all root canal sealers exhibited some toxicity. Therefore, even though newer sealers have been developed owing to their excellent biocompatibility, the cytotoxicity of sealers remains a problem [8]. The biocompatibility of new sealers available in the market is not well documented. However, AH-Plus is a common comparative resin-based sealers demonstrated cytotoxicty to different cells [9].

Well-root St is a new root canal sealant made of calcium silicate that has been shown to precipitate a layer of hydroxyapatite on its surface, possibly creating a mineral connection with dentin tissue [10]. A coating of hydroxyapatite has been shown to precipitate on the surface of Well-root St, a new calcium silicate-based root canal sealer that may build a mineral link with dentin tissue [11]. The same phrase is used to describe their advantageous effects on cell plasticity, such as stimulation of periodontal ligament stem cells (PDLSCs) to differentiate into osteo/odonto/cementogenic cells, which may enhance the healing of periapical wounds [12].

Traditional root canal treatment with sealers did not produce an impermeable seal; thus, novel obturation materials and techniques have been developed over the years to obtain the best seal possible. A recently created silicone-based cold-filling sealant, called GuttaFlow bioseal, contains bioactive glass and GP powder. According to the company, GP and bioactive glass can be mixed to create surface-bound hydroxyapatite crystals. Calcium and silicate, are both encourage tissue regeneration and may have therapeutic properties [13].

The biocompatibility of endodontic sealers can be assessed by implanting tubes with test materials in rat tissues. This process is well-defined, simple to follow, and reproducible [14, 15].

Poor reporting in animal research has an impact on the development of therapies, and irreproducible results can spark the entire field of study or lead to clinical trials that subject patients to interventions that are unlikely to be helpful. The fact that animal experiments are underpowered and use too few animals to produce reliable results is a recurrent concern regarding their validity [16]. Therefore, to ensure the validity of our findings, we employed a sufficient number of animals in our investigation.

The impact of sealers on macrophages should be considered [17] because of the significance of these cells and their role in both innate and acquired immunity as well as inflammation. The majority of cells in the periradicular tissues are macrophages [18].

Immunohistochemistry (IHC) analysis is a method for identifying the presence and placement of proteins in tissue slices. It provides for the monitoring of processes in the context of intact tissue but is quantitatively less sensitive than immunoassays such as western blotting or enzyme-linked immunosorbent assay (ELISA). IHC analysis can be used to assess the presence and location of proteins in the tissue slices. It enables the monitoring of processes in the setting of intact tissue; however, it is less sensitive than immunoassays, such as western blotting or ELISA. This is especially helpful in managing and forecasting the course of diseases such as cancer. In general, IHC and microscopy offer a "big picture" that can help in understanding the data collected using other approaches [19, 20].

This study used histological and immunohistochemical analyses to evaluate the biocompatibility of three different root canal sealers (Well-Root St, GuttaFlow Bioseal, and AH-Plus) after implantation in the subcutaneous tissues of a rat model at three different time intervals. The null hypothesis of this study contends that there is no distinction between the tested sealers.

## Materials and methods

### Sample size calculation

The sample size was planned using (G* Power) computerized software, guided by the results of a published study [21], producing a minimum of 84 samples (18 samples per group). The sample size was increased to (21 per group) for animals that may have been lost during the experiment (effect size = 0.46, Pooled SD = 183.76, alpha (α) = 0.05 and Power).

### Randomization and blinding

Due to the double-blind nature of the study, neither the data gatherer nor the data analyst who carried out the statistical analysis was not aware of the sealer that was employed for the implantation tests. Tissue samples and sealers were classified into several groups and subgroups using a coded number from the allocator. The allocator randomly divided and sort the rats used in the implantation test into groups and subgroups with coded numbers. Computer software was used to create random sequences (http://www.random.org/) [22]. Appropriate sealer tubes and tissue samples were then coded.

### Selection of samples

In this study, 84 healthy Wistar- 90 days old Wistar-Albino male rats weighing 250 g each were used. The animals were randomly allocated into four groups according to the type of sealer used, with 21 animals in each group.

### Preparation of implants

Eighty four sterilized polyethylene tubes (Aldawlia Co., Egypt) measuring 0.9 mm in diameter and 10 mm in length. Four equally sized groups of the tubes were formed (*n* = 21). A specific root canal sealer (Table 1) was packed into 63 tubes using a no. 3 endodontic plugger with a 0.70 mm tip size under aseptic conditions after being mixed according to the manufacturer's instructions. Group A consisted of Well-Root St (Vericom, South Korea), GuttaFlow Bioseal (Coltene Whaledent, Switzerl) Group B, and AH-Plus (Dentsply, Mallefer, Germany) Group C. 21 sterilized and empty implanted polyethylene tubes formed the control group [23].

**Table 1:** Sealers used in this study

Material	Composition	Manufacturer
**Well Root St**	- Calcium silicate - Zinc oxide - Fillers	Vericom, South Korea
**Guttaflow Bioseal**	- Gutta percha powder - Polydimethylsiloxane with nanometer-sized silver particles	Coltene Whaledent, Switzerland
**AH Plus**	Paste A: - Epoxy resin, - Calcium tungstate, - Zirconium oxide, - Aerosil, Iron oxide Paste B: - Adamantane amine, - N,N¢-Dibenzoyl-5-oxanonane diamine-1,9-TCD-diamine, - Calcium tungstate, - Zirconium oxide, - Silicone oil, - Aerosil	Dentsply Mallefer, Germany

### Sedation and anesthesia

The Suez Canal University Faculty of Dentistry animal house is where the experiment was carried out, and the animals were kept in individual cages. Rats were sprayed with neocidol (diazinon) at a concentration of 6/1000 mL of water. Food was avoided 6 h before the procedure. Fifteen minutes prior to general anaesthesia, midazolam 0.25 mg/kg was intramuscularly injected into each animal to cause sedation. The rats were put to sleep using intraperitoneal injections of xylazine (7 mg/kg) (Adwia, Egypt) and ketamine HCL (50 mg/kg) from Trittau, Germany [24].

### Surgical procedures

When the rats were unresponsive, their dorsal skin was shaved and cleaned with povidone-iodine. A 15-mm-long incision in the skin was made using a knife, and a subcutaneous pocket was created with blunt dissection on either side of the incision. The tubes were placed into both filled and empty pockets. After the tubes were inserted, the wound was surgically closed with a single resorbable suture (4/0 silk suture) and cleansed with povidone-iodine [23].

### Post-operative care

After the tubes were implanted, the test animals were placed in the designated cages. Throughout the study period, they consumed water and regular feed (solid food). After 7, 15, and 30 days, samples were collected and seven animals from each group were sacrificed. The animals were sacrificed via cervical dislocation (after anesthesia) in accordance with the recommendations of the ethics committee, as authorized by the Faculty of Dentistry at Suez Canal University in Egypt, Approval No. (211–2019) [23].

### Histological evaluation

Tissue samples from the regions where the tubes were located were embedded in paraffin and fixed in 10% formalin for 24 h. To view the general tissue structure, sections were cut at a thickness of 5 µm, mounted on slides, and stained with hematoxylin and eosin. Three observers with a minimum of three years of expertise used a light microscope to examine the tissue surrounding the implants to look for signs of vascular alterations, the presence of inflammatory cells, and the presence and placement of fibrous tissue.

### Immunohistochemical analysis

For immunohistochemical examination, an indirect streptavidin–biotin-peroxidase approach was used. Samples of 3 mm thickness were cut from the paraffin blocks. Tissue sections were rehydrated after deparaffinization. The slides were treated with hydrogen peroxide for 10–15 min, followed by two washes with buffer to eliminate non-specific background signal staining caused by endogenous peroxidase. To re-establish the antigenic sites and dissolve the cross-linking, the samples were washed and then given an antigenic recuperation treatment with 0.5% pepsin (pH 1.8) for 30 min at 37 °C. Primary CD3 and CD68 antibodies from Thermo Fisher ScientificTM, Lab VisionTM, USA, were used for 18 h at 4 °C at a 1:100 dilution. The samples were cleaned four times in buffer before treatment for 10–15 min at room temperature with biotinylated goat anti-polyvalent antibodies.

After washing in buffer four more times, the sample was exposed to streptavidin peroxidase solution for 10 min at room temperature. Once the desired reaction was achieved, the samples were added to the chromogenic substrate diaminobenzidine (Dab). The sections were washed and counterstained with Mayer's hematoxylin for 8 min. Densitometry analysis was carried out using Video Test Morphology ® software (Russia) for computer-assisted digital image analysis [25].

### Computer assisted digital image analysis

The CD3 marker for T lymphocyte detection and CD68 marker for macrophage detection were used in the immunohistochemistry analysis in this study. The slides were prepared and examined.

### Slide imaging and digitization

With a 40X lens and an Olympus digital camera mounted on an Olympus microscope (Leica DM100, Leica Microsystems, Germany). The generated photos were examined on a PC with an Intel Core I7 processor using the VideoTest Morphology® program (Russia), which uses a dedicated built-in stain quantification technique. Five random fields from each of the two slides created for each subgroup were evaluated.

### The software routine of quantification includes

Depending on the color of the target stains, a picture is acquired from the camera using a u-tech® frame grabber and then enhanced in terms of color tones. The region of interest (ROI), also known as the target stain area, is represented by a binary mask created by the threshold of the picture at the level of the desired stain hue range. Applying the ROI to the stain density measurement procedure. Each result was exported as an XLS file and displayed as a share of the total area.

### Statistical analysis

The data's normal distribution was examined using the Shapiro–Wilk test. The information was parametric and disseminated normally. The mean and standard deviation were included in the descriptive statistics of the lymphocyte and macrophage infiltration. The infiltration of lymphocytes and macrophages was compared between groups and over the course of observation using a one-way ANOVA. Between groups, there was a significant difference in the mean percentage of infiltrating lymphocytes and macrophages (*P* < 0.001), as well as between observation times (*P* < 0.001). (Statistical Package for the Social Sciences, version 25) was used to analyze the data.

## Results

### Histological examination

**Fig. 1:**
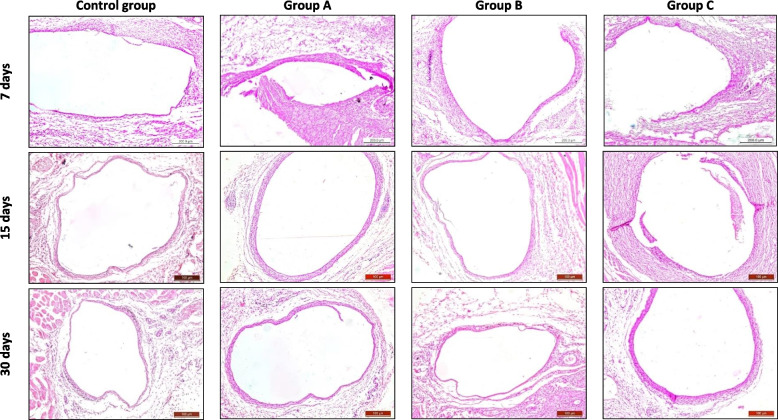
Photomicrograph of subcutaneous tissues representing inflammation intensity and thickness of fibrous capsule of control group, group A: Well-Root St, group B: GuttaFlow Bioseal and group C: AH-Plus after 7, 15 and 30 days at 10X magnification

Histological examination of subcutaneous tissues representing inflammation intensity and thickness of the fibrous capsule of all groups after 7, 15, and 30 days at 10X magnification, (Fig. 1).

After 7 days, the control group and group B were infiltrated with neutrophils, lymphocytes, and macrophages with a moderate inflammatory reaction and formation of a fibrous capsule. Groups A and C showed severe inflammatory cell infiltration comprising lymphocytes and macrophages present in a thick fibrous capsule.

After 15 days, the control group was infiltrated with macrophages, plasma cells, and lymphocytes with a mild inflammatory reaction, the fibrous capsule was thinner, and there were no giant cells or areas of necrosis. In groups A and B, the intensity of inflammation and thickness of the fibrous capsule were reduced. Group C showed severe inflammatory infiltration, and increased thickness and organization of the fibrous capsule.

After 30 days, the control group showed a thinner fibrous capsule and less inflammatory reaction than that at 15 days. Groups A and B showed significant reductions in inflammatory cell infiltration and thinning of the fibrous capsule. Group C showed a reduction in the number of inflammatory cells and a thinner fibrous capsule than those at 15 days.

### Immunohistochemical results

#### CD3 marker results (lymphocytes infiltration)

**Fig. 2:**
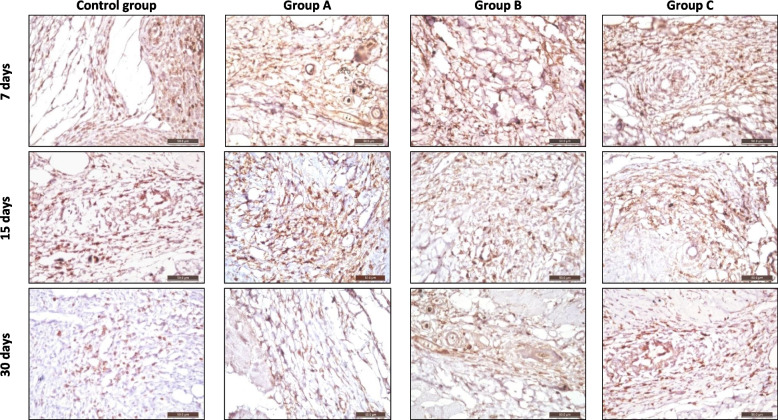
Photomicrograph showing histological sections of subcutaneous tissues at the interface between the host tissue and the implant stained with (CD3 marker) representing lymphocyte infiltration indicated inflammation intensity of the control group, group A: Well-Root St, group B: GuttaFlow Bioseal, and group C: AH-Plus after 7, 15, and 30 days at 40X magnification

Histological sections of subcutaneous tissues at the interface between the host tissue and the implant stained with (CD3 marker) representing lymphocyte infiltration, indicated inflammation intensity in all the groups after 7, 15, and 30 days at 40X magnification, (Fig. 2).

After 7 days, the control and B groups showed moderate positive staining, indicating moderate lymphocyte infiltration. Group A showed moderate-to-strong positive staining. However, group C showed strong positive staining reaction,

After 15 days, the control group and group B showed weak to moderate positive staining reaction indicating reduction in Lymphocyte infiltration. Group A showed moderately positive staining. Group C showed moderate to strong positive staining reaction.

After 30 days, the control group and group B showed weak positive staining. Group A showed weak to moderate positive staining. Group C showed moderate positive staining reaction.

#### CD68 marker results (macrophages infiltration)

**Fig. 3:**
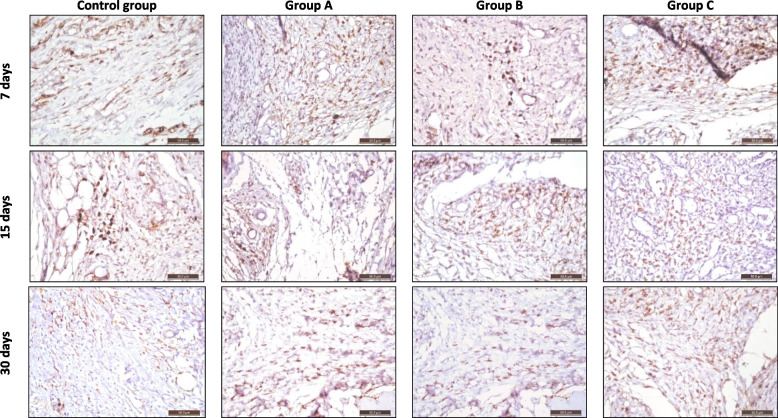
Photomicrograph showing histological sections of subcutaneous tissues at the interface between the host tissue and the implant stained with (CD68 marker) representing macrophage infiltration indicated inflammation intensity of the control group, group A: Well-Root St, group B: GuttaFlow Bioseal, and group C: AH-Plus after 7, 15, and 30 days at 40X magnification

Histological sections of subcutaneous tissues at the interface between the host tissue and the implant stained with (CD68 marker) representing macrophage infiltration indicated inflammation intensity in all the groups after 7, 15, and 30 days at 40X magnification, (Fig. 3).

After 7 days, the control and B groups showed moderate positive staining, indicating moderate macrophage infiltration. Group A showed a moderate positive staining reaction and group C showed a strong positive staining reaction.

After 15 days, the control, B, and C groups showed weak to moderate positive staining, indicating weak to moderate macrophage infiltration. Group C showed moderate positive staining.

After 30 days, the control and B groups exhibited weak positive staining. Group A showed weak to moderate positive staining reaction. Group C showed moderate positive staining reaction.

### Lymphocytes infiltration

#### Comparison of mean percentage of lymphocytes infiltration between groups (Table 2)

**Table 2:** Comparison of mean percentages of lymphocytes infiltrate between groups and observation times

	7 day		15 days		30 days		
	**X**	**SD**	**X**	**SD**	**X**	**SD**	
Control group	9.81 **A, a**	1.09	5.10 **A, b**	1.07	2.19 **A, c**	0.62	< .001*
Group A	17.04 **B, a**	4.58	8.36 **B, b**	0.56	3.59 **A, c**	0.56	< .001*
Group B	15.89 **B, a**	1.25	5.20 **A, b**	0.77	3.00 **A, c**	0.81	< .001*
Group C	22.10 **C, a**	1.91	15.30 **C, b**	1.40	4.10 **A, c**	0.86	< .001*
*P* value	< .001*		< .001*		**.32**		

^*^p value is significant at 5% level. Different lower case letters in the same raw showed a significant difference between each 2 observation times (Bonferroni test, *p* < .05). Different upper case letters in the same column indicate significant difference between each 2 groups (Bonferroni test, *p* < .05). Same letters showed a non-significant difference between each 2 observation times or each 2 groups (Bonferroni test, *p* > .05)

At 7 and 15 days, there was a significant difference in the mean percentage of lymphocyte infiltration between the groups, and the highest mean percentage of lymphocyte infiltration was noted in group C, followed by group A, group B, and the lowest mean percentage was noted in the control group.

At day 30, there was no significant difference in the mean percentage of lymphocyte infiltration between the groups. At 7 days, there was a significant difference between each 2 groups except between group A and group B. At 15 days, there was a significant difference between each 2 groups except between control group and group B.

### Macrophages infiltration

#### Comparison of mean percentage of macrophages infiltration between groups (Table 3)

**Table 3:** Comparison of mean percentages of macrophages infiltrate between groups and observation times

	7 day		15 days		30 days		
	**X**	**SD**	**X**	**SD**	**X**	**SD**	
Control group	5.30 A, a	0.60	3.00 A, b	1.15	1.00 A, c	0.20	< .001*
Group A	7.30 B, a	0.94	4.30 B, b	0.86	1.74 A, c	0.57	< .001*
Group B	5.49 A, a	1.44	4.00 B, b	0.81	1.46 A, c	0.45	< .001*
Group C	19.35 C, a	0.98	4.34 B, b	0.33	4.11 B, b	0.48	< .001*
*P* value	< .001*		**.047**		< .001*		

^*^P value is significant at 5% level. Different lower-case letters in the same raw showed a significant difference between each 2 observation times (Bonferroni test, *p* < .05). Different;fupper-case letters in the same column indicate significant difference between each 2 groups (Bonferroni test, *p* < .05). Same letters showed a non-significant difference between each 2 observation times or each 2 groups (Bonferroni test, *p* > .05)

After 7, 15, and 30 days, there was a significant difference in the mean percentage of macrophage infiltration between the groups; the highest mean percentage of macrophage infiltration was noted in group C, followed by group A, group B, and the lowest mean percentage was noted in the control group.

After 7 days, there was a significant difference between each 2 groups except between group control and group B.

After 15 days, no significant differences were noted among groups A, B, and C. There was a significant difference between control group and other groups.

After 30 days, no significant difference was noted between control group, group A, and group B. There was a significant difference between group C and other groups.

## Discussion

One of the most effective methods for determining the type and course of endodontic sealer-induced local responses is subcutaneous tissue implantation in a rat model. To assess the biocompatibility, the intensity and length of the inflammatory response were assessed. To determine the duration of the reaction in the tissue, histological analysis of the response to materials should be performed [26].

In this study, answers were assessed after 7, 15, and 30 days. The initial moderate reaction in the control samples was most likely caused by surgical stress because a sterile polyethylene tube is a semi-inert object that does not promote inflammation [27]. On day 30, a healthy connective tissue capsule was observed around the implants, and the responses around the control tubes were diminished.

Due to the fact that all polyethylene tubes were inserted before the material had time to set, there was a higher risk of biological breakdown [28]. To facilitate material extrusion into the surrounding tissues, sealers that had just been mixed were poured into the tubes. The inner diameter of the tubes served as the minimal diameter of the contact region, which is less beneficial than typical clinical settings with a smaller foramen of built canals (between 0.3 and 0.6 mm). Because sealer particles immediately interact with the tissues surrounding the open ends of the tubes, responses that are worse than those that have been clinically described may develop [29].

Hematoxylin and eosin are among the most popular tissue stains used for histology. The most frequently employed gold standard stain for making medical diagnoses is this stain [30]. Other specific stains and processes, such as immunohistochemical analysis, are used because staining does not always provide adequate contrast to identify all tissues, cellular structures, or chemical compound distributions [31]. Antigens on lymphocytes and macrophages were identified in this study using immunohistochemical techniques [32]. In this study, CD3 and CD68 were used as markers for lymphocyte and macrophage infiltration, respectively. Immunohistochemical staining was performed using antibodies that recognized the target protein. Antibodies have a high degree of specificity; thus, they bind only to the protein of interest in the tissue segment. Then, either chromogenic detection (an enzyme conjugated to the antibody cleaves a substrate to produce a colored precipitate at the location of the protein) or fluorescent detection (a fluorophore conjugated to the antibody and visible using fluorescence microscopy) is used to visualize the antibody-antigen interaction [33, 34].

Macrophages are a vital component of granulation tissue. These cells clear the healing zone of any remaining cells, fibrin, or other debris. Other inflammatory cells, including neutrophils, lymphocytes, and mast cells, also proliferate and migrate if sufficient chemotactic cues are present. These processes are involved in the migration and proliferation of fibroblasts during tissue repair. However, the inflammatory response brought on by root canal fillings may impair the repair procedure rather than play an intended defensive role because of toxic inflammatory components [35].

The control group showed a modest inflammatory response after 7 days in the current experimental setup. The Well-Root St group demonstrated substantial inflammatory cell infiltration consisting of lymphocytes and macrophages, which led to the establishment of a thick fibrous capsule. The tissue was infiltrated by neutrophils, lymphocytes, and macrophages, which led to the formation of a fibrous capsule. The GuttaFlow Bioseal group displayed a mild inflammatory response, with lymphocytes and macrophages predominating in dense fibrous capsules. Greater than the control, Well-Root St., and GuttaFlow Bioseal groups, the AH-Plus group experienced a strong inflammatory reaction that was predominantly made up of lymphocytes and macrophages in a dense fibrous capsule.

On day 15, the fibrous capsule in the control group was thinner, and the inflammatory response was less severe. The tissue also contains lymphocytes, plasma cells, and macrophages. No obvious large cells or patches of necrosis were observed. The Well-Root St group also showed a decrease in the degree of inflammation and the thickness of the fibrous capsule. While the AH-Plus group displayed increased inflammatory infiltration and a thicker and more structured fibrous capsule, the GuttaFlow Bioseal group displayed less inflammation and a thinner fibrous capsule.

The capsule in the control group was thinner after 30 days than on day 15, and the inflammatory response was lower. Fibrous capsules in the Well-Root St group were thinner and contained fewer inflammatory cells. The group treated with GuttaFlow Bioseal showed a marked decrease in inflammatory infiltration and fibrous capsule thinning. Compared with the 15-day group, the AH-Plus group showed fewer inflammatory cells and a thinner fibrous capsule.

Day 7 inflammatory reaction ratings were the highest for AH-Plus, which was used as the reference because its biological characteristics are well known [36]. Its high concentration of amines, which are used to speed up the setting time, may be responsible for its severe early toxicity [37]. In addition to cytotoxicity [38], the release of bisphenol A diglycidyl ether, a mutagenic component of resin-based goods, may further increase the initial inflammatory response. However, after the initial inflammatory response, both sealers healed faster. These results corroborate earlier research and show that, compared to other endodontic sealers, epoxy resin-based materials cause a longer-lasting and more powerful chronic inflammatory response [39].

According to Simsek et al. [23] and Grecca et al., the AH-Plus group had macrophages in contact with the material after 7 days [40]. According to this study, the initial stage of interaction with connective tissue occurred when AH-Plus was the most aggressive.

Stem cells from rat pulp and subcutaneous tissues performed better in cell cultures when GuttaFlow Bioseal was used [36]. Ca2 + and OH-, which are bioactive ions, are released and serve as epigenetic cues that promote tissue mineralization [37]. Additionally, GuttaFlow Bioseal demonstrated an alkalinizing effect that aided the biological response and sped up tissue repair [38].

The inclusion of bioglass particles in the GuttaFlow Bioseal formulation, as opposed to GuttaFlow Bioseal, enhanced the interaction of bioactive ions and other cement elements with the surrounding media, with no appreciable increase in dissolution (0.11%) [41]. In contrast, cell migration, proliferation, and adhesion are stimulated in vitro by bioactive ions (silicon, calcium, and phosphorus) [36].

Santos et al. [42] found that GuttaFlow Bioseal only moderately increased the inflammatory responses during all observation periods. The initial inflammatory responses of GuttaFlow Bioseal and AH-Plus subsided by day 30, and all of the evaluated sealers showed outstanding biocompatibility.

In the cytotoxicity test, Well-Root St. showed a middle-ground inflammatory response between GuttaFlow Bioseal and AH-Plus. Few studies on the behavior of this recently discovered calcium silicate-based sealer in rat subcutaneous tissue have been published.

All the tested sealers were biocompatible, as evidenced by the fact that the intensity of the inflammatory reactions diminished over time.

In contrast, Gomes et al. [43] found that after 7 days, the tissue had improved in structure in all sealer groups and had become infiltrated with long-term inflammatory cells such as macrophages, lymphocytes, and plasma cells. AH-Plus was used to identify developing fibrous capsules.

The null hypothesis that there was no difference in the biocompatibility of the tested sealers was partially rejected in light of this investigation.

## Conclusion

Guttaflow Bioseal, followed by Well Root St. and AH Plus, was the most appropriate product for the testing conditions. Both the GuttaFlow Bioseal and control groups exhibited minimal inflammation throughout the monitoring period. After subcutaneous tissue implantation in a rat model, the initial inflammatory reactions to Well-Root St and AH-Plus disappeared by day 30, and all tested sealers displayed outstanding biocompatibility. Within the limitations of this study More research is required to describe the physiological response to cytotoxic effects on periapical tissues over time, given the limitations of this study. It was determined that slight adverse effects on the physical and sealing characteristics were not harmful to human cells. Therefore, these new bioactive materials have the potential to improve the effectiveness of endodontic treatments, safeguard tooth roots, and lengthen the lifespan of the teeth.

## Data Availability

The datasets generated and analyzed during the current study are not publicly available due to (ownership of data) but are available from the corresponding author on reasonable request.
